# Changes in Race Performance During the Underwater Phases of a 200 m Bi-Fins Race Simulation After Application of Respiratory Muscle Training—A Case Study in the Current World Record Holder

**DOI:** 10.3390/sports12110306

**Published:** 2024-11-12

**Authors:** Tomáš Michalica, Jakub Březina, Marek Polach, Dennis-Peter Born, Jiří Mališ, Zbyněk Svozil, Eva Kociánová

**Affiliations:** 1 Department of Social Science in Kinanthropology, Faculty of Physical Culture, Palacký University Olomouc, 779 00 Olomouc, Czech Republic; tomas.michalica01@upol.cz (T.M.); jakub.brezina02@upol.cz (J.B.); marek@umimplavat.cz (M.P.); ibn@seznam.cz (J.M.); zbynek.svozil@upol.cz (Z.S.); 2 Umimplavat.cz, Analysis and Consultation for Swimming Technique and Race Performance, 198 00 Praha, Czech Republic; 3 Swiss Swimming Federation, Swiss Development Hub for Strength and Conditioning in Swimming, CH-3048 Worblaufen, Switzerland; dennis.born@swiss-aquatics.ch; 4 Department at University Hospital Olomouc, Palacký University Olomouc, 779 00 Olomouc, Czech Republic

**Keywords:** swim training, video analysis, finswimming performance, respiratory muscle training, underwater phases

## Abstract

Maximal athletic performance can be limited by various factors, including restricted respiratory function. These limitations can be mitigated through targeted respiratory muscle training, as supported by numerous studies. However, the full potential of respiratory training in competitive finswimming has not been fully investigated. This case study aims to evaluate the effects of eight-week respiratory muscle training (RMT) on performance variability during the underwater phases of a 200 m bi-fins race simulation in an elite finswimmer (current world record holder and multiple world championship medalist). Performance variability was assessed based on pre-test, inter-test, and post-test data. Each measurement included pulmonary function and swim performance evaluations. In this study, underwater performance parameters, such as distance, time, velocity, and number of kicks, were assessed using video analysis synchronized with race timing and evaluated using the Dartfish software. The swimmer followed a 28-day training program with an Airofit PRO™ respiratory trainer between tests, with daily sessions targeting both inspiratory and expiratory muscles. The training involved 6–10 min of targeted exercises per day. Significant improvements were observed in Wilcoxon’s paired-sample test between the pre-test and post-test results in terms of underwater distance (*p* = 0.012; d = 1.26), underwater time (*p* = 0.012; d = 1.26), and number of underwater kicks (*p* = 0.043; d = 1.01), resulting in a 14.23% longer underwater distance, 14.08% longer underwater time, and 14.94% increase in underwater kicks. Despite the increased distance and time, underwater velocity remained stable, indicating improved underwater performance efficiency. Despite some improvements, it is not possible to conclude that respiratory muscle training (RMT) can contribute to improved finswimming performance during the underwater phases of a 200 m bi-fins race simulation in this particular athlete’s case. Further research with a larger sample size is necessary to fully understand the impact of RMT on finswimming performance.

## 1. Introduction

The aim of sports training is to prepare athletes to achieve their maximum performance and surpass their competitors. Athletic performance can be significantly affected by respiratory system limitations [1]. Fatigue in respiratory muscles limits ventilation, increases perceived exertion, and disrupts the coordination between breathing and movement, eventually decreasing performance [2,3,4,5]. Respiratory muscle training (RMT) is widely used in physiotherapy and pulmonary rehabilitation to improve respiratory function and strength [6,7,8,9,10]. However, it is also considered a modern training method that can be applied in sports practice to enhance athletic performance [11,12]. Respiratory muscle training (RMT) has demonstrated a positive impact on performance across various sports by improving not only the efficiency of respiratory muscles and delaying their fatigue but also by increasing endurance and strength. In endurance-based dry-land sports such as cycling [13,14] and rowing [15,16], studies have shown that RMT leads to significant increases in inspiratory muscle strength and endurance, resulting in improved oxygen utilization and sustained performance during high-intensity prolonged efforts. In the field of aquatic sports, the application of RMT remains a highly debated issue [17]. However, several studies suggest that integrating RMT into training programs can improve respiratory parameters and swim performance [18,19,20,21].

Recent studies [22,23] emphasize the potential for performance enhancement in swimming, especially in acyclic phases (start and turns), where swimmers are allowed to swim underwater for up to 15 m. These sections of the race allow for higher velocities due to reduced wave drag [24] as well as higher initial velocities attained after the dive [25,26] or wall push-offs [27]. Changes in performance are primarily observed during underwater phases, often exhibited as a shortened underwater distance, fewer kicks, or reduced velocity [22,23]. Underwater distances vary from 5 to 14 m, depending on the discipline [28,29,30], and swimmers typically utilize a sinusoidal motion consisting of up-kicks and down-kicks [31]. The importance of the underwater phase has recently also been confirmed in finswimming in the bi-fins category [32]. The authors of one study compared the performance parameters between Czech elite finswimmers and their world-class counterparts. They identified key differences primarily in underwater velocity and underwater distance, with Czech swimmers achieving a longer breakout but significantly lower speed in the bi-fins disciplines of 100 and 200 m. Therefore, it is crucial to consider the role of breath holding and the associated discomfort due to the lack of oxygen, which may limit the benefits of a long underwater phase. Despite this, no studies have yet examined changes in race parameters during simulated or real race conditions following supplementary RMT application. In this case study, we assessed the impact of an eight-week RMT program on underwater phase performance during a 200 m bi-fins race simulation. An Airofit PRO™ device was used as a tool to facilitate RMT, with a focus on understanding its effects on the athlete’s performance rather than evaluating the device itself.

## 2. Materials and Methods

### 2.1. Participant

An elite female finswimmer (age 24 years; height 186 cm; body mass 89.8 kg) who is a current world record holder (400 m bi-fins race) and a medalist in the world championships in 2022 (100 and 200 m bi-fins races) participated in this case study. The seasonal training program consisted of swim sessions (7 ± 1.5 h per week) and dry-land sessions (2 ± 1.5 h per week) within the framework of traditional linear periodization. The volume of swimming training during the selected period ranged from 16.8 ± 3.4 km per week. Throughout the chosen training mesocycles, no long-lasting specific training blocks with a focus on the development of hypoxia were implemented in the swimmer’s preparation. The participant (n = 1) agreed with all the testing procedures and provided informed consent in compliance with the ethical committee and procedures of Palacký University Olomouc (under reference number 57/2022). This case study was conducted in line with the Code of Ethics of the World Medical Association (Declaration of Helsinki).

### 2.2. Procedure

**Figure 1:**
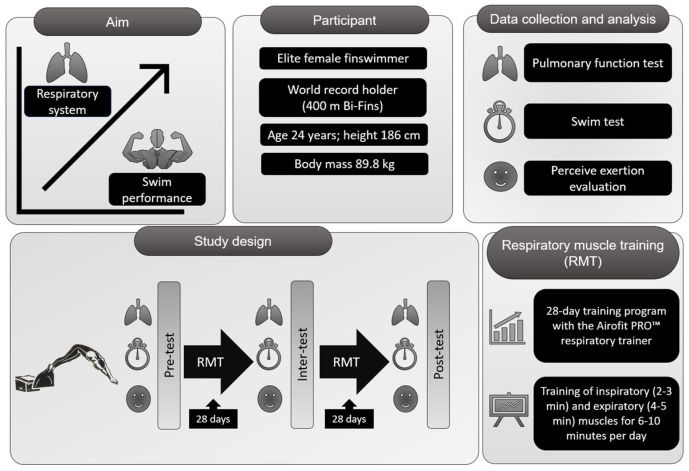
Experimental protocol.

Pre-, inter-, and post-test data were used to evaluate the results in this case study. The testing process consisted of three major parts (pulmonary function tests and a swim test). The swim test (200 m bi-fins race simulation) was performed in a 25 m pool (27.3 ± 0.3 °C). The test was preceded by a standard warm-up divided into a general part (without fins) and a specific part (with fins). The race simulation was conducted 30 min after the warm-up and according to international CMAS rules [33]. The swim test was assessed using a standardized video analysis methodological procedure [34]. Immediately after each race simulation, the swimmer’s perceived level of breathlessness was subjectively assessed on a modified Borg dyspnea 0–10 scale [35]. The intervention in the swimmer’s training process was implemented by integrating supplementary RMT using an Airofit PRO™ respiratory trainer (Figure 1). This device is a small, portable, lightweight, and non-invasive pressure meter with a rubber flange mouthpiece designed for respiratory muscle training. The Airofit PRO™ E-unit contains pressure sensors and a Bluetooth transmitter, allowing for the measurement of breathing patterns and their visualization on a mobile device through the Airofit PRO™ Sport smart application. Additionally, the Airofit PRO™ respiratory trainer provides adjustable airflow resistance [36]. The level of resistance is set based on the initial measurement, the selected type of training program, the duration of the training unit, and its intensity. For purposes of this case study, a 28-day training program for competitive swimming was chosen, which included daily diagnostics and training of inspiratory (2–3 min) and expiratory (4–5 min) muscles for 6–10 min per day. The lung test recorded her accessible vital lung capacity (5.00 ± 1.13 L), maximal inspiratory pressure (105 ± 7.01 cmH_2_O), and maximal expiratory pressure (91 ± 12.578 cmH_2_O). Following this, the swimmer received a visualization in the app with instructions for setting resistance to airflow during respiration. Inhalation air resistance (grade A–F) ranged from 20–140 cmH_2_O/(L/s), and exhalation air resistance (grade 1–6) ranged from 30–200 cmH_2_O/(L/s). Throughout the 28-day periodization protocol, progressive overload in the form of increasing resistance to airflow during respiration was monitored and adjusted based on the swimmer’s current condition. However, the results of studies observed in competitive swimmers were obtained after 6–8 weeks [19,37]. Therefore, the 28-day training program was repeated after the intermediate test. The validity of the Airofit PRO™ respiratory trainer was confirmed by the study outlined in [38]. The Airofit PRO™ training system was designed in accordance with the Medical Device Directive—MDD 2007/47/EC.

### 2.3. Data Collection and Analysis

#### 2.3.1. Swim Test

A camera (Sony FDR-AX700, Tokyo, Japan) was positioned 12 m above the water level, and its optical axis was perpendicular to the direction of swimming, in the middle of the pool. Video footage was recorded in mp4 format (full HD, 1920 × 1080, 50 fps) and subsequently analyzed using the Dartfish software (Live S, Fribourg, Switzerland). Selected phases of the simulated race (individual distances travelled under and above water) were assessed when the swimmer’s head passed marks visible on the lane ropes. Before each race simulation, the accuracy of all markers was re-measured and controlled using a measuring tape. A flashed light signal indicated the start of the race simulation and was synchronized with the camera. The first wall contact of the fin determined the 25 m split times during each lap of the test [22,23]. Simultaneously, the split times as well as the final time were controlled by 3 other persons using hand stopwatches (Casio, HS-80TW-1EF, Tokio, Japan), which were only used to double-check the data of the video analysis but were not used for the statistical analysis. During each underwater phase, the following parameters were assessed: underwater distance [m], underwater time [s], underwater velocity [m.s^−1^], and number of underwater kicks. As an additional control parameter, individual surface velocity [m.s^−1^] was assessed to determine whether the expected changes in the underwater phase also negatively or positively affected the above-water phase.

#### 2.3.2. Pulmonary Function Test

The Geratherm Respiratory DIFFUSTIK device (Geratherm Respiratory GmbH, Bad Kissingen, Germany) with diagnostic software BLUE CHERRY^®^ (V 1.3.0.1) was used to measure, evaluate, and display the results of each individual tests. Following selected respiratory parameters were assessed: Vital Capacity (VC), Expiratory Reserve Volume (ERV), Inspiratory Capacity (IC), Forced Expiratory Vital Capacity (FVCex), Forced Expiratory Volume in one second (FEV1), Peak Expiratory Flow (PEF), Maximal Inspiratory Pressure (PImax), Maximal Expiratory Pressure (PEmax), The Index of Respiratory Work, used for assessing muscle fatigue (TTmus).

#### 2.3.3. Perceived Exertion Evaluation

As an additional evaluation (after the swim test only) of perceived breathlessness, the modified Borg dyspnea scale (MBS) was used, which represents a modified Borg scale for the subjective evaluation of the degree of perceived breathlessness, chest pain, and lower limb discomfort [39]. It is a categorical scale with a numerical rating ranging from 0 to 10, where 0 (as a breathlessness rating) corresponds to the sensation of normal breathing (absence of breathlessness), and 10 corresponds to the subject’s maximum possible sensation of breathlessness. Since the perception of exercise-induced breathlessness depends on the stimulus to which the athlete was exposed, the assessment using the Borg scale should ideally be performed at the same workload [40].

#### 2.3.4. Statistical Analysis

Descriptive statistics was used for data analysis in this case study. The data were processed using the STATISTICA software version 31.4.0.14 (Stat-Soft Inc., Tulsa, OK, USA). To verify the accuracy between repeated measurements, two other analysts simultaneously assessed this dataset of this case study. To evaluate the variability in performance during the underwater phases, a non-parametric Wilcoxon paired-sample test for two dependent samples was used. The level of statistical significance was set at α = 0.05. To determine whether the intervention had a real practical effect, Cohen’s d was used with the corresponding range scale as follows: d ≥ 0.80 = large effect; d (0.50–0.80) = medium effect; d (0.20–0.50) = small effect [41].

## 3. Results

### 3.1. Swim Test

The swim test results demonstrate various changes in total swim time and underwater performance parameters. While there was a slight overall decrease in swim time from the pre- to post-test phases, underwater distance and time showed a gradual increase across all test phases. Additionally, underwater velocity and surface water velocity displayed mixed patterns, with decreases from the pre- to inter-test phases, followed by slight increases towards the post-test phase. The number of underwater kicks increased during the inter- and post-test phases (Table 1).

Except for underwater velocity, a significant difference (*p* < 0.001) and large effect size (d ≥ 0.80) were found in all tested kinematic parameters. Underwater distance significantly increased between the pre-test and post-test phases (*p* = 0.012), with changes observed both from the pre-test to inter-test and from inter-test to post-test phases. Underwater time also showed significant increases (*p* = 0.049; *p* = 0.012) between the different test phases. The number of underwater kicks significantly increased (*p* = 0.043) from the pre-test phase to the post-test phase (Table 2).

**Figure 2:**
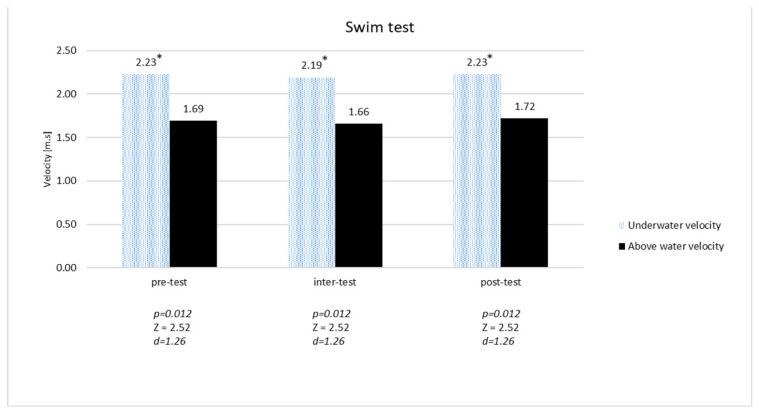
Underwater and surface velocity during pre-, inter-, and post-test phases. * = statistically significant.

Comparing the underwater and surface velocity, a significant difference (*p* = 0.012) and large effect (d = 1.26) was found from the pre- through inter- to post-test phases (Figure 2).

### 3.2. Respiratory Parameters

The results of the pulmonary function test indicate significant changes across the pre-test, inter-test, and post-test phases. Several parameters, such as VC, IC, and FEV1, showed overall increases from the pre- to post-test phases, with the most significant changes occurring between the inter- and post-test phases. In contrast, ERV and FVCex exhibited decreases across the different test phases, with ERV showing a considerable drop after the inter-test phase. Moreover, PEF and TTmus displayed notable improvements during the test phases, while a consistent pattern was observed for PMmax and PEmax, where an initial increase was followed by a slight decrease. The RPE of dyspnea showed a decrease between the pre-test (9) and inter-test (8) phases. The ratio of the values between the inter-test (8) and post-test (8) phases remained unchanged (Table 3).

## 4. Discussion

**Figure A1:**
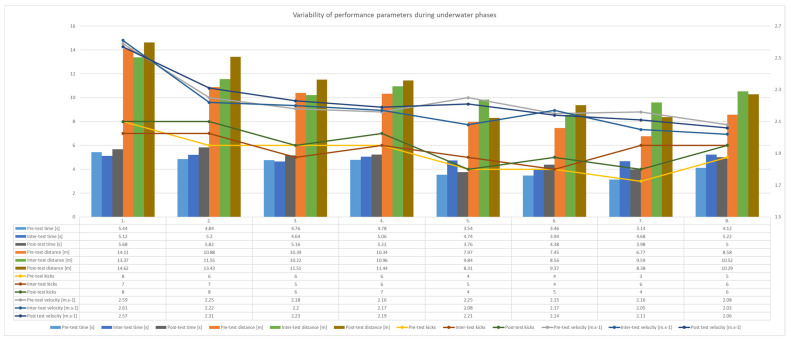
Variability in performance parameters during underwater phases.

The purpose of the present case study was to evaluate the variability in simulated swimming performance in the underwater phases in an elite finswimmer, who is the current world record holder and multiple world championship medalist, after applying an eight-week respiratory training program (Figure A1). The significant findings regarding underwater distance, time, and the number of underwater kicks highlight the practical utility of RMT in enhancing finswimming performance. The progressive increase in underwater distance by 14.23%, coupled with a 14.94% increase in underwater kicks, suggests that RMT facilitates greater efficiency during critical race segments, such as turns and underwater phases. These findings align with previous studies [22,23,28] that have emphasized the role of underwater phases as pivotal for race outcomes, particularly in acyclic segments like starts and turns. However, extending the underwater phase carries the risk of increased physiological fatigue in swimmers due to excessively high hypoxic stimulation [27,42]. Therefore, the distance travelled underwater should be evaluated together with the underwater velocity, which is considered a more relevant parameter for underwater phase assessment [32,43]. A key distinction of this case study lies in the swimmer’s ability to maintain underwater velocity (2.23 ± 0.15 m.s^−1^), even as both the distance and the number of kicks increased. This ability to sustain speed suggests that the swimmer may have preserved the efficiency of the movement pattern, potentially mitigating the impact of hypoxic stress.

However, it is important to contextualize these results within the broader framework of respiratory muscle training. The increase in maximal inspiratory pressure (PImax) by 33.70% following the eight-week program falls within the improvement ranges of 19–45% reported in previous studies on athletes from various sports (rowers [15,16], cyclists [14], football players [44], and young competitive finswimmers [45]). This increase in inspiratory muscle strength likely contributed to the swimmer’s enhanced ability to maintain breath-hold durations during underwater phases, reducing the physiological strain that would typically accompany such efforts [46]. This physiological strain is further influenced by the hydrostatic pressure of the water acting on the swimmer’s chest, which presents an additional challenge for the respiratory muscles during underwater segments [47,48]. In finswimming, it is also important to consider the effort required for exhalation through the breathing tube (or snorkel), which creates an external load on the expiratory muscles [45]. In this case study, the implementation of RMT led to an increase in expiratory muscle strength, reflected in an overall improvement in PEmax by 11.81%, despite a slight decrease of 2.07% after an initial increase of 14.17%. This improvement may have contributed to greater efficiency during the underwater phases and a reduction in cumulative physiological fatigue, which may have manifested during the swimming segments. Our findings are in agreement with results of [4], which highlight that improvements in PImax and PEmax, as observed in this study, appear to be key in reducing the fatigue of the working respiratory muscles during high-intensity exertion.

The fatigue of the respiratory muscles during maximal exertion in athletes may be caused by increased pulmonary ventilation, which disrupts the optimal breathing pattern and leads to an excessive engagement of accessory respiratory muscles. This process can result in inadequate expansion of the chest wall, reduced tidal volume, and increased breathing frequency [3]. Manipulating these variables can significantly affect the efficiency of swimming technique. Studies have shown that more experienced swimmers tend to consciously regulate their breathing to prevent propulsion movements from being restricted by this process [49,50]. Our findings of a 45.45% decrease in the tension–time index of the inspiratory muscles (TTmus) indicate a reduction in the effort required for breathing, which may contribute to maintaining a higher technical performance level during prolonged exertion. This assertion is further supported by the observed improvement in vital functions (VC and FVCex) by 2.91%, allowing for more efficient oxygen utilization during high-intensity swimming, which can positively impact the overall competitive performance of the swimmer and the swimmer’s subjective perception of dyspnea. These findings align with the results of studies outlined in [18,19]. However, to gain a deeper understanding of the complex nature of the mechanisms underlying the changes in respiratory muscle function in the context of improved swimming performance in competitive finswimming, further research is needed. A precise mechanistic explanation for the observed performance enhancement, like that proposed by [19], exceeds the scope of this case study.

## 5. Limitation

This case study was conducted with the current world record holder and a multiple world championship medalist. While this limits the number of participants and the generalizability of the results, it provides insights into the highest possible level of athletic performance. Future research should focus on a larger sample size, including swimmers of lower performance levels, and should include a control group for comparison. It is also important to note that underwater velocity may be influenced by several factors, including the push-off force after the start and each turn [22,26]. Although the assessment of this force was not included in the present case study, we recommend monitoring this parameter in future research to provide a more comprehensive understanding. Additionally, the use of a 25 m pool rather than a 50 m pool represents a limitation, as international competitions are held in 50 m pools. Future research should aim to validate the findings of this study in a 50 m pool to ensure the results are applicable to competitive race conditions.

Furthermore, it is important to note that swimmers’ performance can be influenced by various external variables, such as training conditions, nutrition, and psychological factors. Future research should emphasize monitoring these variables, as they may have a significant impact on overall swimming performance outcomes. In addition, future studies should take into account the variability in body composition parameters, which can also affect performance in the aquatic environment [51]. A more precise understanding of these factors could provide deeper insights into the complex nature of swimming performance and its determinants, thus enabling more effective training approaches and interventions.

## 6. Conclusions

After an 8-week application of respiratory muscle training (RMT), a progressive increase in underwater distance and kicks was observed while maintaining consistent underwater velocity. For this case study, there was no negative impact on clean swim speed, indicating improved efficiency during the underwater phase. This improvement, coupled with a higher clean swim speed, led to an overall enhancement in swimming performance. Despite some improvements, it is not possible to conclude that respiratory muscle training (RMT) can contribute to improved finswimming performance during the underwater phases of a 200 m bi-fins race simulation in this particular athlete’s case. Further research with a larger sample size is necessary to fully understand the impact of RMT on finswimming performance.

## Figures and Tables

**Table 1:** Swim test—performance parameters.

		Swim				
	Total Swim Time [s]	Underwater Distance [m]	Underwater Time [s]	Underwater Velocity [m.s^−1^]	Underwater Kicks	Surface Water Velocity [m.s^−1^]
		M ± SD	M ± SD	M ± SD	M ± SD	M ± SD
Pre-test	106.72	9.56 ± 2.22	4.26 ± 0.76	2.23 ± 0.15	5.25 ± 1.48	1.69 ± 0.10
Inter-test	107.64	10.58 ± 1.35	4.82 ± 0.40	2.19 ± 0.17	5.75 ± 0.97	1.66 ± 0.09
Post-test	104.30	10.92 ± 2.14	4.86 ± 0.71	2.23 ± 0.15	6.00 ± 1.5	1.72 ± 0.13
% pre-test vs. inter-test	0.86	10.67	13.15	−1.79	10.15	−1.78
% inter-test vs. post-test	−3.10	3.21	0.83	1.83	4.35	3.61
% pre-test vs. post-test	−2.27	14.23	14.08	0	14.94	1.78

Legend: M = median; % = percentage difference; SD = standard deviation.

**Table 2:** Swim test—statistical analysis.

	**Underwater Distance [m]**		
	***p***	**Z**	***d***
Pre-test vs. inter-test	0.068	1.82	0.91
Inter-test vs. post-test	0.401	0.84	0.42
Pre-test vs. post-test	0.012 *	2.52	1.26
	**Underwater time [s]**		
	***p***	**Z**	***d***
Pre-test vs. inter-test	0.049 *	1.96	0.98
Inter-test vs. post-test	0.889	0.14	0.07
Pre-test vs. post-test	0.012 *	2.52	1.26
	**Underwater velocity [m.s^−1^]**		
	***p***	**Z**	***d***
Pre-test vs. inter-test	0.262	1.12	0.56
Inter-test vs. post-test	0.123	1.54	0.77
Pre-test vs. post-test	1.000	0	0
	**Underwater kicks**		
	***p***	**Z**	***d***
Pre-test vs. inter-test	0.345	0.94	0.47
Inter-test vs. post-test	0.554	0.59	0.30
Pre-test vs. post-test	0.043 *	2.02	1.01

*p* = statistically significant; * = statistically significant difference at the *p* < 0.05 level; Z = Wilcoxon’s nonparametric test; *d* = Cohen’s d.

**Table 3:** Pulmonary function test.

	VC [L]	ERV [L]	IC [L]	FVCex [L]	FEV1 [L]	PEF [L/s]	PImax [kPa]	PEmax [kPa]	TTmus
	M	M	M	M	M	M	M	M	M
Pre-test	5.16	1.81	3.35	5.16	4.31	9.13	9.20	12.70	0.11
Inter-test	5.20	1.60	3.52	5.12	4.54	9.55	12.90	14.50	0.07
Post-test	5.31	1.91	3.40	5.31	4.48	10.13	12.30	14.20	0.06
% pre-test vs. inter-test	0.78	−11.60	5.07	−0.78	5.34	4.60	40.22	14.17	−36.36
% inter-test vs. post-test	2.12	19.38	−3.41	3.71	−1.32	6.07	−4.65	−2.07	−14.29
% pre-test vs. post-test	2.91	5.52	1.49	2.91	3.94	10.95	33.70	11.81	−45.45

Legend: M = median; VC = vital capacity; ERV = expiratory reserve volume; IC = inspiratory capacity; FVCex = forced expiratory vital capacity; FEV1 = forced expiratory volume; PEF = peak expiratory flow; PImax = maximal inspiratory pressure; PEmax = maximal expiratory pressure; TTmus = the tension–time index of the inspiratory muscles; % = percentage difference.

## Data Availability

Data from this case study are available from the corresponding author upon reasonable request.
